# Vagally mediated heart rate variability modulates the association between the perceived workload and the Stroop effect on behavioral performance

**DOI:** 10.14814/phy2.70466

**Published:** 2025-07-22

**Authors:** Xiao Yang, Michael Gazzanigo, Fang Fang, Spencer House

**Affiliations:** ^1^ Department of Psychology Old Dominion University Norfolk Virginia USA; ^2^ Research and Infrastructure Service Enterprise Macon & Joan Brock Virginia Health Sciences Old Dominion University Norfolk Virginia USA

**Keywords:** ex‐Gaussian parameters, inhibitory control, stroop effect, vagally mediated heart rate variability, workload

## Abstract

Vagally mediated heart rate variability (vmHRV) reflects top‐down cognitive processes involved in emotion‐cognition integration. Using cognitive control can be effortful and increase negative affect. However, this intrinsic affective component of cognitive control has not been well studied, and the role of vmHRV in the association between subjective experience in using cognitive control and behavioral performance remains unknown. The current study aimed to examine the relationship of vmHRV with cognitive control and perceived workload in a cognitive task. Eighty‐one participants performed the Stroop interference task. Participants rated subjective workload using the NASA Task Load Index (NASA‐TLX) scale for congruent and incongruent trials separately. Moreover, cognitive performance was analyzed with the ex‐Gaussian model, from which the parameters μ and τ were derived to reflect sensorimotor processing and inhibitory control, respectively. Multiple regressions were used to analyze the effects of TLX change score (incongruent–congruent), vmHRV, and their interaction on the Stroop effect. Results showed that vmHRV negatively predicted the Stroop effect on τ. Importantly, vmHRV moderated the association between perceived workload and the Stroop effect on τ. Our findings highlight the role of cardiac vagal control in emotion‐cognition integration and have theoretical and practical implications.

Cognitive and affective processes are intertwined and influence each other at different levels. Although cognition and emotion were historically studied as two distinct aspects of mental life, the integration of cognition and emotion has been supported by accumulating evidence (Damasio, 2003; Ochsner & Gross, 2005; Öhman et al., 2001; Phelps, 2004; Schwarz, 2000). Research on emotion‐cognition integration has focused on how affective components influence information processing. For example, threat‐related, negative stimuli enhance perception and attention through bottom‐up processes (Fox et al., 2001; LeDoux, 1996; Öhman et al., 2001). The facilitation of information processing by emotionally negative stimuli is automatic and reflexive (Öhman et al., 2001; Phelps, 2004). In contrast, when performing a cognitive task that requires high levels of mental effort, human information processing involves controlled processes (Shiffrin & Schneider, 1977). Those controlled activities rely on the function of the prefrontal cortex (PFC), which is also known as cognitive control or supervisory attentional control (Norman & Shallice, 1986).

Cognitive control refers to the mental processes that coordinate thoughts and actions to flexibly adapt to changing goals (Inzlicht et al., 2015; Miller & Cohen, 2001). It is closely related to executive functions—a set of higher‐order cognitive processes that regulate other mental operations, including inhibition, working memory (WM), and cognitive flexibility (Baddeley, 1986; Diamond, 2013). While some researchers use the terms cognitive control and executive functions interchangeably (e.g., Chun & Most, 2021; Menon & D'Esposito, 2022; Smith & Kosslyn, 2007), cognitive control specifically emphasizes the dynamic application of executive functions within a given context (Capuana et al., 2014). In this article, we focus on cognitive control and specify the type of executive functions involved in a task. Cognitive control helps down‐regulate negative affect and manage emotional responses (Ochsner & Gross, 2005). Meanwhile, negative emotions and stress may impair cognitive control (for reviews, see Arnsten, 2009; Joormann & Tanovic, 2015; Shields et al., 2016). However, studies on emotion‐cognition integration have typically considered affective reactivity and cognitive control as separate processes (e.g., Brosschot et al., 2017; Ruiz‐Padial & Thayer, 2014; Toh et al., 2024), which has led to the overlooking of the intrinsic affective component of cognitive control.

According to a model of emotion‐cognition integration, cognitive control may be understood as an affective process (Inzlicht et al., 2015). Cognitive control is initiated when conflicting and discrepant goals induce negative emotions (Carver & Scheier, 2011; Elliot & Devine, 1994; McNaughton & Gray, 2000). Converging evidence from behavioral performance and neuroimaging findings supports the association between cognitive control and aversive affective responses (Botvinick, 2007; Dreisbach & Fischer, 2012; Fritz & Dreisbach, 2013). Moreover, cognitive control is subjectively costly, which is consistent with its emotionally aversive nature (Dixon & Christoff, 2012; Kool et al., 2010, 2013; Westbrook & Braver, 2015).

Of note, cognitive effort describes the degree of cognitive control involved in monitoring and resolving conflicting goals, and it varies across individuals, mirroring trait differences in the affective components of cognitive control (Cacioppo & Petty, 1982; Westbrook et al., 2013; Westbrook & Braver, 2015). The subjective experience of cognitive effort has been linked to activation of the ventromedial prefrontal cortex (vmPFC) and anterior cingulate cortex (ACC; Seamans et al., 2024; Westbrook et al., 2019), and those brain regions are also implicated in negative emotions (Goldin et al., 2008; Mak et al., 2009; Ochsner et al., 2004). Given that cognitive effort reflects subjective experience in cognitive control, examining cognitive effort may help disentangle the intrinsic affective components and mental operations of cognitive control. Studies have reported that excessive perceived cognitive effort contributes to impairments in cognitive performance among older adults (Westbrook et al., 2013) and individuals with depression (Westbrook et al., 2023), schizophrenia (Culbreth et al., 2016), and anorexia nervosa (King et al., 2024).

The subjective aspect of cognitive effort can be evaluated by perceived workload that is defined as the intensity of a task someone experiences and expects within a given amount of time (Wickens, 2008). Perceived workload is commonly measured by self‐report scales, including NASA Task Load Index (NASA‐TLX; Hart & Staveland, 1988). NASA‐TLX assesses the perception of task quantity, complexity, and intensity, and reflects subjective cognitive demands and emotional experience (Hancock & Meshkati, 1988; Hockey, 1997; Wickens, 2008). The scale is sensitive to individual differences in cognitive and affective processes. When performing a task with a given difficulty, TLX scores were rated higher by groups with cognitive impairment and affective disorders (Westbrook et al., 2013, 2023). Perceived workload was also negatively correlated with behavioral performance in the n‐back task (Seidman et al., 2023). Moreover, self‐rated workload exhibited the same pattern of changing with increased n‐back levels as that of task performance (i.e., a coupling of perceived workload and behavioral performance; Seidman et al., 2023), suggesting that there are common top‐down cognitive resources underlying both subjective experience in cognitive control and task performance.

The autonomic nervous system (ANS) plays a critical role in emotion‐cognition integration (Quadt et al., 2022). According to the Neurovisceral Integration Model (NIM), the brain modulates cognitive and affective processes through the ANS (Thayer & Lane, 2000, 2009). While both branches of the ANS (i.e., the sympathetic and parasympathetic nervous systems) have connections with various brain regions, parasympathetic activity, or vagal control, is more often studied in relation to the central autonomic network (CAN; Benarroch & Chang, 1993). Further, the CAN includes vmPFC and ACC, the brain regions involved in the affective components of cognitive control (Jennings et al., 2015; Thayer et al., 2012). Therefore, vagally mediated heart rate variability (vmHRV), mirroring the effect of the vagus nerve on cardiac inter‐beat intervals, is thought to indicate the functioning of the CAN as well as top‐down processes involved in the integration of cognitive control and affective processes (Thayer et al., 2012; Thayer & Lane, 2000, 2009).

At rest, higher levels of vmHRV have been associated with better cognitive control (for reviews, see Arakaki et al., 2023; Forte et al., 2019; Magnon et al., 2022). For example, higher resting vmHRV was linked to faster processing speed and fewer response errors, and those relationships became stronger when the task required cognitive control (Hansen et al., 2004). Further, vmHRV positively predicted performance in cognitive control tasks, including a backward digital span task (Mann et al., 2015), a standard executive function assessment battery (Kimhy et al., 2014), a stop signal task (Colzato & Steenbergen, 2017), a verbal memory task and a Stroop task (Al Hazzouri et al., 2017), and task‐switching (Colzato et al., 2018). Interestingly, unlike the association between vmHRV and cognitive control among healthy individuals, HRV metrics of stroke patients did not show relationships with performance in cognitive control tasks, which indicated that the connection between the ANS and the brain areas supporting cognitive functions was weakened by neurological conditions (Beer et al., 2017). It is possible that the patients in the study (Beer et al., 2017) failed to engage in the cognitive tasks as much as healthy controls did. However, there were no assessments of subjective experience in the study (Beer et al., 2017), which prevented clarifying the role of cognitive effort in task performance.

According to the NIM, high resting vmHRV indicates greater amounts of top‐down resources for cognitive control (Thayer & Lane, 2000, 2009). In contrast, individuals with lower levels of vmHRV have fewer available resources. While it is easy for individuals with high vmHRV to recruit additional cognitive resources when task load increases, those with low HRV need to utilize most resources to maintain a certain level of cognitive performance from the beginning of the task and thus are inflexible to allocate already insufficient resources (Yang et al., 2021). Because the cognitive resources used for performing a task also underlie the perception of workload, low vmHRV individuals may show the stronger association between perceived workload and task performance compared to their high vmHRV counterparts. Put another way, the coupling of subjective experience and behavioral performance would be stronger among individuals with lower levels of cardiac vagal control. Several studies have reported associations between vmHRV and subjective workload measured by the NASA‐TLX. For example, Delliaux et al. (2019) reported that the standard deviation of RR intervals (SDNN) was negatively correlated with NASA‐TLX ratings in a letter recognition task. In simulation tasks, HRV measures, including root mean square of successive differences (RMSSD) and SDNN, were also negatively correlated with NASA‐TLX scores (Alaimo et al., 2020; Wulvik et al., 2019). Moreover, Muth et al. (2012) assessed high frequency (HF) HRV and subjective workload in a simulation task with multiple difficulty levels and found that baseline HF HRV negatively predicted NASA‐TLX scores across all difficulty levels. However, most of these studies were correlational in nature, lacked well‐controlled experimental conditions, and did not systematically investigate task performance. No study has yet examined the possible modulation of the perceived workload‐performance relationship by vmHRV.

Among various mental operations of cognitive control, inhibitory control resolves conflicting goals (Inzlicht et al., 2015) and is of great importance for daily functioning (Diamond, 2013). Compared to other mental operations of cognitive control, inhibitory control has a stronger relationship with vmHRV (Magnon et al., 2022), and it suppresses automatic and impulsive responses and reduces the interference of irrelevant stimuli and thoughts (Aron et al., 2014). Based on the NIM, Brosschot et al. (2017) proposed the Generalized Unsafety Theory of Stress (GUTS) that the default defense/stress response of an organism is tonically active unless the PFC exerts top‐down control to inhibit it (i.e., inhibitory control). Also, the GUTS highlighted the indicative value of vmHRV in the inhibitory processes for top‐down PFC resources (Brosschot et al., 2017; Thayer et al., 2012), which is also consistent with a recent meta‐analysis (Magnon et al., 2022). As pointed out in the meta‐analysis, tasks involving inhibitory control demand higher levels of self‐regulatory effort associated with cardiac vagal control (Magnon et al., 2022).

One cognitive task that has been widely used to examine inhibitory control is the Stroop task (Stroop, 1935). The task presents words printed in different colors and requires subjects to inhibit automatic, prepotent responses and to report semantic information and process the color of letters as the task‐relevant information when interference arises from the mismatches between the word and the color of its letters, that is, incongruent trials (Stroop, 1935; Tucha & Lange, 2004). Compared to congruent trials, subjects took longer response times and exhibited more errors in incongruent Stroop trials, which is known as the Stroop effect (Stroop, 1935). The Stroop effect is robust across the experimental paradigms using different stimuli and responses, as well as various populations (for a review, see MacLeod, 1991). The brain structures, ACC, dorsolateral PFC (dlPFC), and vmPFC, are involved in the Stroop task, which serve to identify conflicts between competing responses, suppress automatic but task‐irrelevant processing, and detect errors and adjust responses, respectively (Carter et al., 2001; MacDonald et al., 2000; Milham et al., 2001). Those neural structures are also involved in cognitive effort (Seamans et al., 2024; Westbrook et al., 2019) and the central regulation of ANS activities (Jennings et al., 2015; Thayer et al., 2012). Research has shown that, in healthy populations, higher resting vmHRV was associated with greater accuracy and faster response times in both congruent and incongruent trials in the standard Stroop task (Spangler et al., 2018) and in a Stroop task involving financial incentives (Capuana et al., 2014). Also, in a modified Stroop task that included an attentional set‐shifting component, resting SDNN and RMSSD were negatively correlated with response times of incongruent trials with different response requirements (to name the color print—inhibition, or to read the color‐word) (Stenfors et al., 2016). Moreover, individuals with high vmHRV tend to exhibit a smaller Stroop effect compared to those with low vmHRV (Forte & Casagrande, 2025). However, in clinical populations, the relationships of Stroop task performance with vmHRV metrics, including HF HRV, SDNN, and RMSSD, appear to be moderated by pathological conditions such as somatic symptom disorder and cardiovascular disease (Huang et al., 2021; Toyofuku et al., 2024). Despite the documented associations between vmHRV and the Stroop effect, how inhibitory control involved in the Stroop task influences subjective experience of cognitive effort and its relationship with ANS indicators of emotion‐cognition integration remains unknown.

The Stroop effect on behavioral performance has various components. Several studies used the ex‐Gaussian model to examine the Stroop effect on different behavioral components (Heathcote et al., 1991; Spieler et al., 1996). As the shape of response times (RTs) is usually skewed, the traditional metrics, for example, mean and median RT, may be misleading and fail to analyze RT variability at the trial‐by‐trial level (Heathcote et al., 1991; Yang et al., 2023). To overcome the limitation of the traditional performance metrics, the ex‐Gaussian model fits the natural distribution of the RT data and teases very slow responses (the exponential tail of the RT distribution) apart from faster trials in the Gaussian (normally distributed) portion of the RT distribution. The components of an ex‐Gaussian distribution indicate separate aspects of behavioral performance. Specifically, while parameters ex‐Gaussian *mu* and *sigma* (μ and σ, represent the mean and the *SD* of the Gaussian component of the RT distribution, respectively) indicate lower‐order sensory‐perceptual processing, the proportion of very slow RTs of the tail, estimated with the parameter *tau* (τ), putatively reflects top‐down control processes (Gmehlin et al., 2016; Leth‐Steensen et al., 2000). Some studies showed that the Stroop interference influenced both parameters μ and τ (Heathcote et al., 1991; Spieler et al., 1996), but other reports indicated that the task conflict (the competition between the processing of different stimuli) and response conflict (the competition between the emission of different responses) differentially prolonged τ and μ, respectively (Steinhauser & Hübner, 2007, 2009). Additionally, ex‐Gaussian τ has been shown to be sensitive to the standard Stroop effect but not to the semantic Stroop effect (White et al., 2016).

## 1 CURRENT STUDY

The current study sought to address the gaps in the aforementioned research. Based on the NIM (Thayer & Lane, 2000, 2009) and the theory of cognitive effort (Westbrook & Braver, 2015), resting vmHRV was expected to modulate the relationship between perceived workload and the performance of inhibitory control in the Stroop task. Specifically, the current study aimed to (1) examine how perceived workload of the Stroop task was related to various components of behavioral performance during the task; (2) investigate whether resting vmHRV is associated with the perceived workload and the performance of the cognitive inhibition task; and (3) test the moderation of vmHRV on the association between perceived workload and task performance. We used a computerized version of the Stroop task (Quesnel & Yang, 2023) and a block design, where the congruent trials and incongruent trials were presented in separate blocks, to assess the perceived workload under the two conditions. To analyze different components of the behavioral performance of the Stroop task, each participant's RT data were fitted using the ex‐Gaussian model, and the ex‐Gaussian parameters were derived for the congruent and incongruent conditions (Yang et al., 2023).

In light of the above literature, we hypothesized that the increase in workload by the Stroop interference would be positively correlated with the reduction in response accuracy and increases in ex‐Gaussian parameters in the incongruent trials compared to congruent trials (*Hypothesis 1*). We also predicted that higher levels of resting vmHRV would be associated with lower levels of the Stroop effect on the behavioral outcomes (incongruent–congruent) during the task (*Hypothesis 2*). Importantly, we hypothesized that the relationship between the perceived workload and the Stroop effect would be stronger among individuals with low vmHRV individuals compared to those with high vmHRV (*Hypothesis 3*). This hypothesis was made to reflect that the sufficient top‐down resources among high vmHRV people can be used to modulate affective processes during the task, which may mask the linear relationship between task‐related subjective experience and task performance.

## 2 METHOD

### 2.1 Participants

Eighty‐one participants (*M*_*age*_ = 20.47 years; *SD* = 6.71 years; 59 female) were recruited from undergraduate psychology courses. The sample size was determined by using G*Power 3.1 (Faul et al., 2009). In the power analysis, the effect size was set at the medium level, *f* ^*2*^ = 0.15, and the significance level and power were set at *α* = 0.05 and (1‐*β*) = 0.8, respectively, which indicated that 77 subjects would be needed. The additional participants were recruited to prevent the impact of attrition. Participants were screened for histories of neurologic and psychiatric illness, cardiovascular disease, and color blindness, and further exclusion criteria included currently taking cardioactive or psychotropic medication. Moreover, none of the participants were smokers. After the recruitment, all participants were required to abstain from alcohol for 12 h and caffeine for 6 h prior to participation to ensure the validity of physiological data (Grant et al., 2023). Participants received course credits for their participation. In the present study, two participants failed to complete the experimental procedure and were thus excluded from the analyses. Additionally, another participant was excluded from the analyses due to equipment failure in physiological recording. Therefore, the final sample consisted of *N* = 78 participants (*M*_*age*_ = 20.53 years; *SD* = 6.92 years; 57 female). The study was approved by the Old Dominion University Institutional Review Board. Written informed consent was obtained from all participants prior to participation.

### 2.2 Task, design, and materials

A modified Stroop task was used to assess cognitive inhibition and sensorimotor processing (MacLeod, 1991; Quesnel & Yang, 2023; Stroop, 1935). Specifically, the task stimuli were the words “GREEN” and “RED” in green or red ink, which were presented on a computer screen. In the congruent condition, the word “GREEN” was displayed as the green letters, and the “RED” as the red letters. On the contrary, in the incongruent condition, the word “GREEN” was displayed as the red letters, whereas the letters in the “RED” were green. Participants were asked to press the corresponding keys on a computer keyboard in response to the color of the letters of the word (rather than the word itself) as quickly as possible.

**FIGURE 1:**
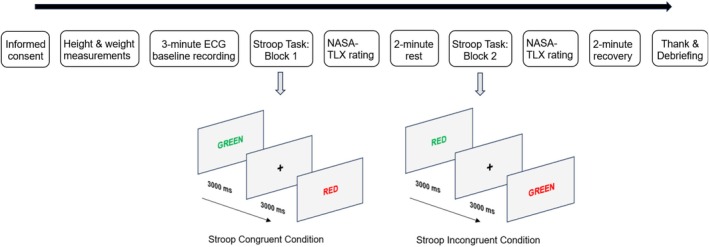
Procedure and stimulus presentation of the study. The order of the congruent and incongruent conditions of the Stroop task was randomly counterbalanced in the first and second blocks. ECG, electrocardiography; NASA‐TLX, NASA task load index.

The congruent and incongruent conditions were delivered as two separate blocks. The order of the two blocks was randomly counterbalanced across participants. Each of the two conditions contained 100 Stroop trials. In a trial, the stimulus was presented for 2500 ms or ended by a detected key pressing response. The inter‐trial interval was 3000 ms. Participants rated their perceived workload after each block. There was a 2‐min rest period between the two blocks of the Stroop task (see Figure 1).

The NASA Task Load Index (NASA‐TLX) was used to assess subjective workload during each block of the Stroop task. The NASA‐TLX is a self‐rated questionnaire of perceived workload and includes six items on a 7‐point scale. In the computer version of the NASA‐TLX, there are increments of high, medium, and low estimates for each point, which generate a 21‐point Likert scale to assess dimensions of workload: mental demand (*How mentally demanding was the task?*), physical demand (*How physically demanding was the task?*), temporal demand (*How hurried or rushed was the pace of the task?*), performance (*How successful were you in accomplishing what you were asked to do?*), perceived effort (*How hard did you have to work to accomplish your level of performance?*), and frustration (*How insecure, discourage, irritated, stressed, and annoyed were you?*), respectively (Hart & Staveland, 1988). The NASA‐TLX has been used to evaluate subjective workload across various tasks in laboratory and real‐life settings, and indicates a good test–retest reliability (Pearson's *r* = 0.81; Hart & Staveland, 1988) and acceptable to good internal consistencies (Noyes & Bruneau, 2007; Rubio et al., 2004; Zheng et al., 2012). In the current study, the NASA‐TLX showed Cronbach's *α* = 0.71, indicating an acceptable reliability.

### 2.3 Procedure

Upon arrival at the laboratory, informed consent was obtained from all participants. Before the experiment, participants' height and weight measurements were taken to compute body mass index (BMI). They were then comfortably seated in the laboratory while physiological recording sensors were attached. To assess cardiac vagal control, participants were instructed to sit still and watch a 180‐s emotionally neutral film that depicted aquatic scenes, which was consistent with the “vanilla baseline” guidelines to evaluate cardiovascular indices at the resting baseline (Jennings et al., 1992). After the baseline measurement, participants were provided with the instructions of the Stroop task and completed 10 practice trials. The first block of the Stroop task began immediately after the practice trials. At the end of the first block, participants rated NASA‐TLX to report subjective workload during the block, and then took a rest for 2 min. Following the rest period, the second block of Stroop trials was delivered, and participants also rated NASA‐TLX at the end of the block. After the completion of the Stroop task and NASA‐TLX assessments, participants were asked to sit still and remain quiet for 2 min as the recovery period. The physiological recording sensors were then removed, and participants were thanked and informed about the purpose of the study (see Figure 1).

### 2.4 Physiological recording and apparatus

The laboratory task and the NASA‐TLX were delivered on a 58‐cm diagonal wide computer screen located 0.6 m in front of the participant. The instructions and stimuli of the Stroop task were presented using the E‐prime 3.0 software (Psychology Software Tools, Pittsburgh, PA).

The BIOPAC MP160 system (BIOPAC Systems Inc., Goleta, CA) was used to collect physiological data, which were digitized at 1000 Hz (16‐bit) and analyzed with BIOPAC AcqKnowledge software 5.0 (BIOPAC Systems Inc., Goleta, CA). Electrocardiography (ECG) was recorded with disposable, pre‐gelled stress‐testing spot electrodes using a modified Lead II configuration (ConMed Andover Medical, Haverhill, MA). Moreover, respiratory activity was measured by a respiration transducer placed around the subject's torso at the thoracic level.

### 2.5 Data reduction

The data of the behavioral performance during the Stroop task included response accuracy and reaction time (RT). Response accuracy was calculated as the percentage of correct responses in all trials of each block. RT data were analyzed using the ex‐Gaussian approach. Specifically, the correct RT trials that were longer than 100 ms in each block were included in the ex‐Gaussian modeling (Yang et al., 2023). Note that slow RT trials were not excluded, which was different from the traditional calculation of the mean or median RT. The short RT trials (<100 ms) were deleted to avoid the confounding effects of guessing on sensorimotor responses, which constituted <0.1% of all data for a participant. This approach is consistent with the guideline (Luce, 1986). The RT distribution of each condition was fitted with the ex‐Gaussian model using the “retimes” package in *R* statistical software (Massidda & Massidda, 2013). To ensure the robustness of the models, bootstrapping with 1000 iterations was used to estimate the ex‐Gaussian parameters μ, σ, and τ (Spangler et al., 2018). Due to the focus on response speed and attentional processes in the present study, μ and τ would serve as the dependent variables in the analysis (Yang et al., 2023; Yang, Herberlein, et al., 2024). In addition, mean RTs were also calculated to validate the ex‐Gaussian approach. As for subjective ratings, the sum of the scores of all six NASA‐TLX items was used to indicate the perceived workload for each block. The change scores of the behavioral and self‐report data were calculated as *Incongruent–Congruent* to indicate the effects of the Stroop interference (MacLeod, 1991; Quesnel & Yang, 2023).

The AcqKnowledge software was used to identify ECG R‐peaks and derive inter‐beat intervals (IBIs) from the raw signals, and trained raters also manually inspected those R‐spikes. The ECG data of all participants met the criterion for further analyses where <5% of signals were influenced by artifacts (Yang & Friedman, 2017; Yang et al., 2019, 2021). VmHRV was quantified as high‐frequency (0.15–0.4 Hz) HRV (HF‐HRV) fast Fourier spectral power (the frequency‐domain metric of cardiac vagal control) and the root mean square successive differences (RMSSD) of R‐R intervals (the time‐domain metric; Berntson et al., 1997), by using Kubios HRV analysis software v2.0 (Biosignal Analysis and Medical Imaging Group, Kuopio, Finland). HF‐HRV values were normalized using a logarithmic transformation with the base of 10 due to the skewed distribution.

Respiration data were acquired to examine gross respiratory artifacts in ECG data and to validate the selection of the frequency band of HRV analyses. Average respiratory frequency of all participants during the resting baseline was within the range of 0.15 to 0.4 Hz.

### 2.6 Analytic approach

To examine the Stroop interference effect, NASA‐TLX scores, response accuracy, the ex‐Gaussian parameters, and mean RTs were submitted to one‐way repeated measures analyses of variance (ANOVA). Moreover, the relations among demographic variables, changes scores of NASA‐TLX, and behavioral performance, and metrics of vmHRV were calculated by Pearson correlations.

Multiple regression models were constructed to investigate the influence of vmHRV and subjective workload on cognitive inhibition, and to test the interaction between vmHRV and workload:

$$\begin{array}{c} \text{∆}\text{Behavioral Performance}=\beta_{0}+\beta_{1}\;\text{∆NASA}-\mathrm{TLX}\\ \;+\beta_{2}\;\mathrm{HF}-\mathrm{HRV}+\beta_{3}\;\text{∆TLX}\times \mathrm{HF}-\mathrm{HRV} \end{array}$$

In the models, ∆Behavioral Performance represents the change score (Incongruent–Congruent) of the behavioral indicator, that is, response accuracy, ex‐Gaussian μ and τ, which was entered into the model as the dependent variable. Each behavioral indicator was examined in a separate model. Workload increase (i.e., NASA‐TLX change score) and HF‐HRV served as the independent variables and were mean‐centered in the regression models. In the equation, the coefficients *β*_0_, *β*_1_, *β*_2_, and *β*_3_ represent the intercept, the effects of the independent variables, and the interaction on the dependent variables, respectively.

All regression models were controlled for age, sex, and BMI, which are known to influence sensorimotor responses and cardiac vagal control. For the sake of simplicity, the covariates were not shown in the equation. In addition, RMSSD was entered into the regression models, as a substitute vmHRV measure, to check the validity of HF‐HRV in reflecting cardiac vagal control. Effect sizes of independent variables were estimated by *R* ^*2*^. Simple slope analysis was applied to analyze significant interaction terms in the regression models (Aiken et al., 1991).

## 3 RESULTS

### 3.1 Descriptive statistics and manipulation check

Mean BMI of the final sample (*N* = 78) was 25.30 (*SD* = 4.31). During the resting baseline, physiological data indicated mean IBI = 722.89 ms (*SD* = 100.49 ms), mean HF‐HRV = 2.58 log ms^2^ (*SD* = 0.50 log ms^2^), mean RMSSD = 32.53 ms (*SD* = 19.42 ms), and respiratory frequency = 0.20 Hz (*SD* = 0.03 Hz).

As shown in Table 1, in the Stroop task, the incongruent condition reduced response accuracy and prolonged mean RTs, the ex‐Gaussian parameters μ and τ, but not σ, compared to the congruent condition. Moreover, participants experienced higher levels of workload under the incongruent condition than those under the congruent condition (see Table 1).

**TABLE 1:** Descriptive Statistics of behavioral performance during the stroop task.

Variable	Congruent condition	Incongruent condition	Unadjusted *F* statistics
Response Accuracy (M, SD)	0.96 (0.09)	0.91 (0.19)	6.39*
Ex‐Gaussian μ (M ms, SD)	451.74 (105.00)	490.56 (123.67)	12.21***
Ex‐Gaussian σ (M ms, SD)	70.82 (37.62)	81.60 (46.77)	3.58
Ex‐Gaussian τ (M ms, SD)	158.96 (81.73)	283.62 (129.26)	110.48***
Response Time (M ms, SD)	596.79 (145.64)	756.98 (204.92)	100.40***
NASA‐TLX score (M, SD)	29.64 (21.26)	38.64 (22.93)	35.01***

*Note*: Behavioral performance and the perceived workload (NASA‐TLX) in the congruent and incongruent conditions of the Stroop task were compared using one‐way repeated measures analysis of variance (ANOVA). The parameters μ, σ, and τ were derived from the ex‐Gaussian modeling.

* *p* < 0.05.

*** *p* < 0.001.

Demographic variables, age, and BMI were correlated with HRV metrics and the change score of τ, respectively (see Table 2). The change in NASA‐TLX was negatively associated with the Stroop effect on response accuracy but positively related to the change in τ (see Table 2). However, HRV metrics were not correlated with any behavioral variables or NASA‐TLX score.

**TABLE 2:** Pearson correlation coefficients among study variables.

Variable	1	2	3	4	5	6	7
Age	–						
2 BMI	0.06	–					
3 ∆Accuracy	0.02	−0.46	–				
4 ∆Ex‐Gaussian μ	−0.08	−0.16	0.19	–			
5 ∆Ex‐Gaussian τ	−0.17	0.36**	−0.43**	0.02	–		
6 ∆NASA‐TLX	0.16	0.10	−0.32**	−0.03	0.38**	–	
7 HF‐HRV	−0.27*	−0.13	0.11	0.05	−0.16	−0.07	–
8 RMSSD	−0.22*	−0.14	0.05	0.01	−0.13	−0.10	0.88**

*Note*: The variables ∆Accuracy, ∆Ex‐Gaussian μ, ∆Ex‐Gaussian τ, and ∆NASA‐TLX were calculated as the change scores of those indices from the Stroop congruent to incongruent condition. HF‐HRV and RMSSD, which were the metrics of heart rate variability and derived from electrocardiography signals.

Abbreviations: HF‐HRV, high frequency heart rate variability; RMSSD, root mean square successive differences.

* *p* < 0.05.

** *p* < 0.01.

### 3.2 Moderation of HRV on association between behavioral performance and workload

The multiple regression analyses are summarized in Table 3. Model 1 tested the effects of NASA‐TLX change score and HF‐HRV and their interaction on the Stroop effect on response accuracy. Consistent with the Pearson correlation, the results of Model 1 showed that greater changes in NASA‐TLX ratings predicted smaller decreases in response accuracy, *β*_1_ = −0.01, *t*(71) = −3.41, *p* = 0.001, *R* ^2^ = 0.11 (see Table 3). However, neither HF‐HRV nor the interaction term predicted response accuracy change.

**TABLE 3:** Summary of the multiple regression models.

Dependent variable	∆NASA‐TLX		HF‐HRV		Interaction term	
	*β*_1_ (*SE*)	*R* ^*2*^	*β*_2_ (*SE*)	*R* ^*2*^	*β*_3_ (*SE*)	*R* ^*2*^
Model 1						
∆Response Accuracy	−0.01 (0.01)**	0.11	0.05 (0.05)	0.01	0.01 (0.01)	0.03
Model 2						
∆Ex‐Gaussian μ	0.07 (0.92)	0.01	1.11 (24.90)	0.01	−0.50 (2.58)	0.01
Model 3						
∆Ex‐Gaussian τ	3.62 (0.78)***	0.19	−49.26 (21.12)*	0.05	−5.44 (2.19)*	0.05

*Note*: The variables ∆Accuracy, ∆Ex‐Gaussian μ, ∆Ex‐Gaussian τ, and ∆NASA‐TLX were calculated as the change scores of those indices between the congruent and incongruent conditions. All models were controlled for age, sex, BMI; the independent variables were mean‐centered in the models; the coefficients in the table are unstandardized.

Abbreviation: HF‐HRV, high frequency heart rate variability.

* *p* < 0.05.

** *p* < 0.01.

*** *p* < 0.001.

The model of the Stroop effect on μ (Model 2) did not indicate any relationship of the change score of μ with NASA‐TLX score or HF‐HRV (see Table 3).

The results of Model 3 showed that the Stroop effect of the ex‐Gaussian parameter τ was positively predicted by NASA‐TLX response, *β*_1_ = 3.62, *t*(71) = 4.65, *p* < 0.001, *R* ^2^ = 0.19, and inversely predicted by HF‐HRV, *β*_2_ = −49.26, *t*(71) = −2.33, *p* = 0.023, *R* ^2^ = 0.05 (see Table 3). Importantly, the interaction between NASA‐TLX change score and HF‐HRV was also associated with the dependent variable, *β*_3_ = −5.44, *t*(71) = −2.48, *p* = 0.015, *R* ^2^ = 0.05 (see Table 3), indicating the moderation of HF‐HRV on the relationship between the Stroop effect on τ and NASA‐TLX score.

To validate Model 3, RMSSD was entered into the model as the substitute vmHRV measure. The results of RMSSD and the original Model 3 were similar, except for that RMSSD did not predict τ change score. Specifically, while RMSSD was not a significant predictor, *β*_2_ = −90, *t*(71) = −1.61, *p* = 0.111, NASA‐TLX and the interaction term still predicted the Stroop effect on τ, *β*_1_ = 3.37, *t*(71) = 4.29, *p* < 0.001, *R* ^2^ = 0.17, and *β*_3_ = −0.09, *t*(71) = −2.01, *p* = 0.048, *R* ^2^ = 0.04 (see the supplemental materials for the complete results of all models).

**FIGURE 2:**
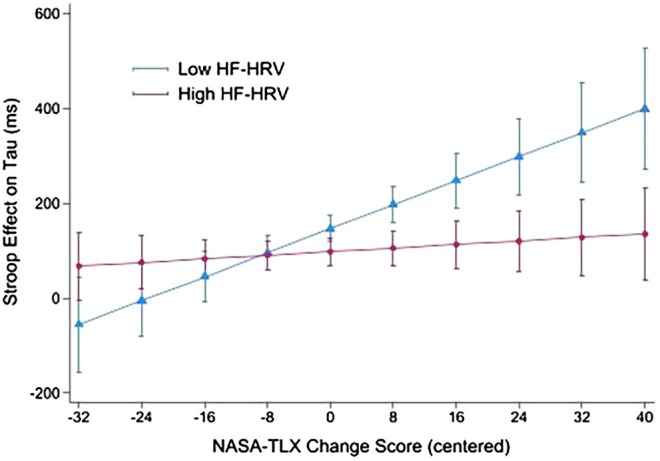
Simple slope analysis of high frequency heart rate variability (HF‐HRV) moderating the relationship between the Stroop interference effect on the ex‐Gaussian parameter tau (τ) and the changes in the perceived workload (mean centered NASA‐TLX change score: Incongruent–Congruent). Low and high HF‐HRV groups represent the observations below‐1 SD and above 1 SD, respectively; the error bars represent 95% C.I. of the bins.

Further, the interaction between NASA‐TLX and HF‐HRV in Model 3 was explored by the simple slope analysis. The results indicated that, among individuals with HF‐HRV below −1 *SD* (the low vmHRV group), the NASA‐TLX change score positively predicted the Stroop effect on τ, *β* = 11.18, *p* = 0.023, whereas that relationship was not evident among individuals with HF‐HRV above +1 *SD* (the high vmHRV group), *β* = 0.80, *p* = 0.634 (see Figure 2).

## 4 DISCUSSION

In the present study, we examined the moderation effects of resting vmHRV on the association between the perceived workload and inhibition performance in a modified Stroop task. In the task, the congruent and incongruent conditions were delivered in separate blocks. The NASA‐TLX was used to assess the perceived workload after each block of the Stroop task. Cardiac vagal control was measured at the resting baseline as vmHRV, which was used to indicate top‐down cognitive resources. Moreover, the behavioral performance was analyzed using the ex‐Gaussian modeling, from which the parameters that reflect different components of information processing were derived. Our hypotheses were partially supported. As predicted, the Stroop effect was evident on response accuracy and the ex‐Gaussian parameters μ and τ. Also, the Stroop interference increased self‐rated workload. Although vmHRV was not related to the perceived workload and task performance, it modulated the association between the perceived workload and the behavioral indices. Additionally, our analyses were controlled for the covariates that might influence cardiac vagal control and behavioral performance, and the results were validated by a substitute vmHRV measure.

Specifically, the first hypothesis regarding the relationship between the Stroop effect and the perceived workload was supported. Response accuracy was lower, and response speed (both ex‐Gaussian parameters and traditional mean RTs) was slower in the incongruent condition than those in the congruent condition, which indicated the Stroop effect on behavioral performance. The present results are in line with the previous findings and demonstrate the robustness of the Stroop effect (Stroop, 1935; Tucha & Lange, 2004). The results of the ex‐Gaussian modeling, the parameters μ and τ, but not σ, were prolonged in the incongruent condition, suggesting that the version of the Stroop task in the present study involves both task conflict and response conflict (Steinhauser & Hübner, 2009). In other words, the Stroop effect was attributed not only to the suppression of the automatic response but also to inhibitory control over the entire stimulus–response pathway for the semantic meaning of the word. In cognitive tasks that do not involve top‐down cognitive processes, ex‐Gaussian τ is thought to indicate attentional lapses and failures in sustaining attention (Spangler et al., 2018, 2021). Unlike those pure sensorimotor tasks, the Stroop effect on τ reflects the cognitive expense of inhibition (Yang et al., 2023). This interpretation of the results of τ is also consistent with the absence of the Stroop effect on σ, in that the variability in RTs may indicate the stability of attentional processes and remained at a similar level in the congruent and incongruent conditions.

It is worth noting that although the ex‐Gaussian approach addresses the issues of traditional RT metrics, the method has its own limitations. As Schmiedek et al. (2007) pointed out, ex‐Gaussian modeling has the problem of parameter dependency, which arises during the data‐fitting procedure. Correlations among estimated ex‐Gaussian parameters may impact the validity of further analyses of the parameters (Schmiedek et al., 2007). More importantly, the ex‐Gaussian distribution is a descriptive model and does not provide conclusive evidence of the cognitive processes underlying its parameters (Matzke & Wagenmakers, 2009). Therefore, converging evidence is needed to support the interpretation of μ and τ in the present study. For example, other computational models have been used to supplement the ex‐Gaussian modeling (Matzke & Wagenmakers, 2009; Schmiedek et al., 2007) and a model tuning method was proposed to improve it (Moret‐Tatay et al., 2018). However, in addition to the reported relationship between τ and cognitive control (Spangler et al., 2018, 2021, 2022; Steinhauser & Hübner, 2007, 2009; White et al., 2016; Yang et al., 2023; Yang, Herberlein, et al., 2024), the present findings of ex‐Gaussian parameter τ suggest its association with top‐down control (see also the later discussion on vmHRV).

Further, processing conflicting information in the incongruent Stroop trials required inhibitory control and increased NASA‐TLX scores. The change in NASA‐TLX ratings was associated with the effects of the Stroop interference on response accuracy and τ (see Table 2), which suggests a mutual relationship between the perceived workload and task performance. Specifically, the items in the NASA‐TLX measure mental demand, perceived performance and effort, and negative affect, which were directly influenced by the poorer performance in the incongruent condition, including slower RTs and more response errors. Meanwhile, elevated perceived workload indicated the demand for more cognitive resources to respond to incongruent Stroop trials. Therefore, with the limited amount of resources, the behavioral performance was impaired among individuals who reported greater changes in NASA‐TLX scores (the stronger Stroop effect). However, a causal relationship was not established between the perceived workload and the performance in the Stroop task. Future studies are expected to explore the temporal sequence of the two domains of cognitive inhibition. In fact, the relationship between the perceived workload and cognitive performance is subject to the influences of individual differences and psychopathology. For instance, higher levels of metacognitive abilities lead to more accurate estimated performance (Fleming & Dolan, 2012); the same level of behavioral performance is associated with greater perceived workload in the groups with attention‐deficit/hyperactivity disorder (Högstedt et al., 2023), anxiety disorders (Eysenck et al., 2007), and depression (Murugesan et al., 2022), compared to healthy individuals.

Contrary to the second hypothesis regarding vmHRV and task performance, there were no relationships between vmHRV and behavioral indices, based on the unadjusted correlation coefficients (see Table 2). Previous studies reported that vmHRV was inversely associated with the ex‐Gaussian parameter τ of the RT distributions in the Flanker task (Spangler et al., 2021) and the Stroop task (Spangler et al., 2018). The present results seem to be inconsistent with those studies (Spangler et al., 2018, 2021). However, there are differences in methodology among the studies, which may contribute to the inconsistency of the findings. Spangler et al. (2018, 2021) did not separate congruent and incongruent trials into different blocks and modeled the RT distributions of all trials. The parameter τ in those RT distributions primarily reflected attentional processes (Spangler et al., 2018, 2021) but was not directly influenced by inhibitory control (the difference in the behavioral performance between the congruent and incongruent condition). The authors might have chosen that approach due to inadequate numbers of total RT trials in the studies (Spangler et al., 2018, 2021). In contrast, we used the block design and included a greater number of trials compared to the previous studies, which allowed the ex‐Gaussian modeling for the RT distributions of the congruent and incongruent conditions separately. Therefore, the Stroop effect on τ (the change score τ from the congruent condition to incongruent condition) was more likely to mirror inhibitory control as opposed to attentional lapses.

A closer examination of the regression model (Model 3) revealed that, after controlling for the covariates, high vmHRV predicted less effect of the Stroop interference on τ. This result aligns with the studies reporting the positive relationship between resting vmHRV and top‐down resources (Spangler et al., 2018, 2021, 2022). Although the model parameters in the present study and those prior studies (Spangler et al., 2018, 2021, 2022) capture different aspects of cognitive control, vmHRV has been shown to be reliably related to cognitive processes that require the PFC resources. In the present study, the relationship between vmHRV and τ was evident only after controlling for sex, age, and BMI, suggesting those noncognitive factors potentially influence the relationship and should be considered in future studies focusing on HRV and cognitive functions.

According to a recent meta‐analysis (Magnon et al., 2022), the presence and strength of the association between vmHRV and cognitive functioning depend on the metrics of vmHRV and the selection of cognitive tasks. Specifically, respiratory sinus arrhythmia (RSA) has shown stronger relationships with cognitive processes compared to HF‐HRV and RMSSD, and vmHRV is more associated with cognitive inhibition than with loading working memory (Magnon et al., 2022). Along with the meta‐analysis (Magnon et al., 2022), our findings of vmHRV and cognitive performance suggest that cognitive effort and cognitive load are distinct aspects of information processing. Magnon et al. (2022) summarized that vmHRV may be more sensitive to performance on tasks involving self‐regulation and flexible cognitive processes, compared to the tasks that primarily rely on working memory and possibly induce mental stress. Similarly, vmHRV was positively related to self‐regulatory effort in a food choosing task, which suggested a need to differentiate between self‐regulation and stress (Segerstrom & Nes, 2007). That is, an effortful cognitive task may not necessarily be stressful; vice versa. Cardiac vagal control is likely to reflect effortful components as opposed to stressful components of cognitive processes.

The third hypothesis was supported by the results. Our study is among the first to show the moderation of vmHRV on the association between subjective experience and behavioral performance (the Stroop effect on τ) in a cognitive control task. The influences of trait‐level differences in cardiac vagal control on the regulatory processes of reflexes and behavior have been reported in recent studies (Melzig et al., 2009; Ruiz‐Padial & Thayer, 2014; Yang et al., 2021). The present finding adds to this emerging literature and supports the GUTS (Brosschot et al., 2017), suggesting that vmHRV indicates the cognitive resources for the coupling of cognitive and affective processes. Specifically, among individuals with low levels of resting vmHRV, cognitive resources are limited and will be allocated to immediate environmental demands. In the present study, those demands were inhibitory control required by the Stroop task. Consequently, low vmHRV individuals' NASA‐TLX scores (including perceived cognitive demands, effort, and performance, and negative affect) were inadequately regulated, which may resemble the elevated ratings of task difficulty and cognitive load among people with depression (Gotlib & Joormann, 2010; Seidman et al., 2023). Poorer performance led to higher NASA‐TLX ratings among low vmHRV individuals. On the contrary, greater amounts of cognitive resources, as indicated by high levels of resting vmHRV, were sufficient for inhibiting the Stroop effect and coping with negative affect, which resulted in unaffected NASA‐TLX scores by the task performance. Therefore, uncoupled perceived workload and inhibition performance indicate flexibility in allocating top‐down cognitive resources.

## 5 IMPLICATIONS

Our findings of vmHRV provide the support for the NIM (Thayer & Lane, 2000, 2009) and the GUTS (Brosschot et al., 2017), suggesting that vmHRV serves as a reliable index of top‐down control processes. More importantly, top‐down cognitive resources can not only be used to modulate other cognitive processes but also influence the intrinsic affective component of cognitive control. In that regard, the current findings integrate affective and cognitive processes at the level of metacognition, which may help understand the role of cognitive control in psychological and behavioral disorders, especially at preclinical and early stages of the diseases.

Given the central role of cognitive control in emotion regulation, our study has practical implications for psychopathology. Emotion dysregulation is considered as a fundamental risk factor of various forms of psychopathology (Ochsner & Gross, 2005), and Beauchaine and Thayer (2015) proposed resting vmHRV as a transdiagnostic marker of psychopathology. Reduced vmHRV and dysregulated affective processes have both been linked to depression (Kemp et al., 2010), anxiety (Friedman & Thayer, 1998), schizophrenia (Montaquila et al., 2015), and posttraumatic stress disorder (Cohen et al., 2000). While high vmHRV buffered negative affective experiences in the cognitively demanding task, the low vmHRV individuals in the present study were sensitive to the level of workload, which is similar to the dysregulated emotions implicated in mental disorders. Relatedly, the Vagal Tank Theory (VTT) proposes cardiac vagal control as an indicator of the mobilization of self‐regulatory resources (Laborde et al., 2018), which is supported by emerging empirical evidence (e.g., De Leon Sautu & Gonzalez, 2024; Prell et al., 2025; Wei et al., 2024). According to the VTT, higher resting vmHRV reflects a “fuller tank” of cardiac vagal control, which predicts better cognitive functioning and more favorable health outcomes, which align with the results of the present study. Beyond resting cardiac vagal control, the theory also posits that vmHRV reactivity and recovery reflect the dynamic processes of vagal resource ‘depletion’ and ‘replenishment’ (Laborde et al., 2018). Thus, future research should explore the dynamic interaction between vmHRV responses and self‐regulatory effort.

Importantly, cardiac vagal control is modifiable, and vmHRV can be enhanced by several approaches, including slow‐paced breathing (for a review, see Laborde et al., 2022), exercise (for a review, see Routledge et al., 2010), the diving reflex (for a review, see Ackermann et al., 2023), and non‐invasive brain stimulation (Schmaußer et al., 2022). Moreover, exposure to low‐illuminance warm‐colored light may increase vmHRV (for a review, see Martins et al., 2025). Accordingly, the target of general preventions and treatments of mental disorders should include those strategies to upregulate vmHRV and improve emotion regulation.

Additionally, the present findings highlight individual differences in monitoring and improving human performance. For example, HRV has been used as a physiological index of mental workload in driving (Matthews et al., 2010), automation (Ting et al., 2009), human‐computer interaction (Kaufmann et al., 2012), and cognitive performance in virtual reality (Alam et al., 2023). In the applied settings, HRV metrics are usually used without specifying their physiological mechanisms and are interpreted incorrectly (Hughes et al., 2019). There is a general agreement that most HRV metrics only inform on cardiac vagal activity (Reyes del Paso et al., 2013; Shaffer & Ginsberg, 2017). However, human factors researchers and practitioners are still using low frequency (LF) HRV and LF/HF ratio to indicate sympathetic activation and predict cognitive stress and human performance (Hughes et al., 2019). This notion regarding LF HRV and LF/HF ratio should be rejected in future research. According to the NIM (Thayer & Lane, 2000, 2009), vmHRV should be selected as the primary indicator of cognitive resources. The present findings suggest that vmHRV not only predicts cognitive performance but also indicates subjective workload, which should be considered in the study of how users interact with devices and systems as well as the customization of automation that optimizes human performance.

## 6 LIMITATIONS, FUTURE DIRECTIONS, AND CONCLUSION

Our findings need to be evaluated in the light of several limitations. First, the NASA‐TLX measures multiple aspects of workload, which are heterogeneous. Although the present study showed an acceptable level of internal consistency among the items of the NASA‐TLX, those items reflect different dimensions of subjective experience during the task. Future studies are expected to develop tools that focus on a single dimension of workload and are suitable for specific tasks (Rubio et al., 2004). Next, as mentioned in earlier sections, although ex‐Gaussian modeling has been used as a solution to the problems of traditional RT metrics, it has certain limitations, including its descriptive nature and parameter dependency. Schmiedek et al. (2007) suggested that the interpretations of the ex‐Gaussian parameters are not straightforward, and the ex‐Gaussian parameters are usually correlated with each other due to the parameters being estimated for individual subjects' data. Thus, additional computational models and neuroimaging data are needed to confirm the present findings. Third, while the interaction term in Model 3 (on the ex‐Gaussian τ) was statistically significant, the effect size was relatively small. In the present study, all regression models were controlled for age, sex, and BMI, so it is possible that the covariates accounted for a certain portion of variance in the models. However, the moderation effect in the present results should still be interpreted with caution, and our findings need to be replicated in future studies. Fourth and relatedly, our sample consisted of healthy young adults, and thus the present results may be difficult to generalize to other populations. It is possible that, due to impairments in the cardiovascular and nervous systems, the relationships of HRV with cognitive performance and perceived workload may be altered among older adults and patients with cardiovascular disease and neurological disorders (Beer et al., 2017). Therefore, our findings should be replicated in clinical samples with psychopathological conditions. Additionally, there are sex differences in vmHRV and its relationships with affective processes and cognitive performance (Koenig & Thayer, 2016; Thayer et al., 2012; Yang, Chaney, et al., 2024). Therefore, our findings should be replicated in gender‐balanced samples, and sex differences should be tested with those samples in future studies. Last, the longer duration of ECG recording should be used to assess vmHRV and other HRV metrics, and vmHRV reactivity and recovery should be examined in relation to perceived workload. We followed the recommendation of the minimal length of ECG recording for HRV power spectral analysis (Berntson et al., 1997). However, the Task Force (1996) suggested the 5‐min recording for short‐term HRV measurements to standardize the results across different studies. Although the present study focused on resting vmHRV due to methodological factors in the study design, vmHRV reactivity and recovery in cognitive tasks are informative and should be examined in future research (Laborde et al., 2018). A recent guideline (Quigley et al., 2024) emphasized the importance of accounting for ECG signal stationarity, motor responses, posture, respiration, and characteristics of cognitive tasks, as these can affect the validity of HRV measurements during active conditions. These possible confounding factors should be considered and controlled in future studies examining dynamic changes in vmHRV.

In sum, the current study is among the first to report the moderation of cardiac vagal control on the association between cognitive performance and perceived workload. We used the ex‐Gaussian modeling to separate different cognitive processes involved in the Stroop task. In the analyses, we controlled for covariates and validated the results using a substitute vmHRV measure. The strengths of our study outweigh its weaknesses. Theoretically, the present findings provide further support for the NIM (Thayer & Lane, 2000, 2009), the GUTS (Brosschot et al., 2017), and the VTT (Laborde et al., 2018), integrate cognitive effort into the NIM, and contribute to better understandings of cognitive control in the context of emotion–cognition interaction. Further, the present study has practical implications for the prevention and treatment of psychopathology as well as the improvement of human performance in applied settings.

## FUNDING INFORMATION

The study was not supported by any funding.

## CONFLICT OF INTEREST STATEMENT

There are no conflicts of interest.

## ETHICS STATEMENT

This study was conducted in accordance with the Declaration of Helsinki and approved by ODU Institutional Review Board. Informed consent was obtained from all subjects involved in this study.

## Supporting information


Data S1.

## Data Availability

The raw data supporting the conclusions of this article will be made available by the authors on request.
