# Supplementary material for: Vagally mediated heart rate variability modulates the association between the perceived workload and the Stroop effect on behavioral performance
Source: Physiol Rep. 2025 Jul 22;13(14):e70466. doi: 10.14814/phy2.70466 (PMC12280812; doi:10.14814/phy2.70466)
Supplement: Supplementary file 1 — Data S1. [file PHY2-13-e70466-s001.docx]

**Supplemental Materials**

**Table A. Coefficients of the Variables in Model 1**

| **Dependent**  **Variable** | **Independent Variable** | **Coefficient (β)** | **Standard Error** | ***p*-value** | | ***R^2^* change** | |  |
| --- | --- | --- | --- | --- | --- | --- | --- | --- |
| *∆Response Accuracy* | |  |  |  |  | |  |  |
|  | ∆NASA-TLX | -0.01 | 0.01 | .001 | 0.11 | | | |
|  | HF-HRV | 0.05 | 0.05 | .293 | 0.01 | | | |
|  | Interaction Term | 0.01 | 0.01 | .068 | 0.03 | | | |
|  | Age (covariate) | 0.01 | 0.01 | .257 | 0.01 | | | |
|  | Sex (covariate) | -0.03 | 0.05 | .526 | 0.01 | | | |
|  | BMI (covariate) | -0.02 | 0.01 | <.001 | 0.16 | | | |

*Note*: HF-HRV = High frequency heart rate variability; BMI = Body mass index; Interaction Term represents the interaction between ∆NASA-TLX and HF-HRV; sex was dummy coded with female as the reference.

**Table B. Coefficients of the Variables in Model 2**

| **Dependent**  **Variable** | **Independent Variable** | **Coefficient (β)** | **Standard Error** | ***p*-value** | | ***R^2^* change** | |  |
| --- | --- | --- | --- | --- | --- | --- | --- | --- |
| *∆Ex-Gaussian μ* | |  |  |  |  | |  |  |
|  | ∆NASA-TLX | 0.07 | 0.92 | .938 | 0.01 | | | |
|  | HF-HRV | 1.11 | 24.90 | .965 | 0.01 | | | |
|  | Interaction Term | -0.50 | 2.58 | .848 | 0.01 | | | |
|  | Age (covariate) | -0.79 | 1.79 | .660 | 0.01 | | | |
|  | Sex (covariate) | -7.55 | 26.63 | .778 | 0.01 | | | |
|  | BMI (covariate) | -3.59 | 2.74 | .195 | 0.02 | | | |

*Note*: HF-HRV = High frequency heart rate variability; BMI = Body mass index; Interaction Term represents the interaction between ∆NASA-TLX and HF-HRV; sex was dummy coded with female as the reference.

**Table C. Coefficients of the Variables in Model 3**

| **Dependent**  **Variable** | **Independent Variable** | **Coefficient (β)** | **Standard Error** | ***p*-value** | | ***R^2^* change** | |  |
| --- | --- | --- | --- | --- | --- | --- | --- | --- |
| *∆Ex-Gaussian τ* | |  |  |  |  | |  |  |
|  | ∆NASA-TLX | 3.62 | 0.78 | <.001 | 0.19 | | | |
|  | HF-HRV | -49.26 | 21.12 | .023 | 0.05 | | | |
|  | Interaction Term | -5.44 | 2.19 | .015 | 0.05 | | | |
|  | Age (covariate) | -4.45 | 1.52 | .005 | 0.07 | | | |
|  | Sex (covariate) | -1.89 | 22.59 | .934 | 0.01 | | | |
|  | BMI (covariate) | -6.70 | 2.33 | .005 | 0.07 | | | |

*Note*: HF-HRV = High frequency heart rate variability; BMI = Body mass index; Interaction Term represents the interaction between ∆NASA-TLX and HF-HRV; sex was dummy coded with female as the reference.

**Table D. Coefficients of the Variables in Model 3 with RMSSD**

| **Dependent**  **Variable** | **Independent Variable** | **Coefficient (β)** | **Standard Error** | ***p*-value** | | ***R^2^* change** | |  |
| --- | --- | --- | --- | --- | --- | --- | --- | --- |
| *∆Ex-Gaussian τ* | |  |  |  |  | |  |  |
|  | ∆NASA-TLX | 3.37 | 0.79 | <.001 | 0.17 | | | |
|  | RMSSD | -0.90 | 0.56 | .111 | 0.02 | | | |
|  | Interaction Term | -0.09 | 0.05 | .048 | 0.04 | | | |
|  | Age (covariate) | -4.24 | 1.55 | .008 | 0.07 | | | |
|  | Sex (covariate) | 1.07 | 23.26 | .963 | 0.01 | | | |
|  | BMI (covariate) | 7.17 | 2.38 | .004 | 0.08 | | | |

*Note*: RMSSD = Root mean square successive differences; BMI = Body mass index; Interaction Term represents the interaction between ∆NASA-TLX and RMSSD; sex was dummy coded with female as the reference.
